# Neural correlates of suppressing and imagining future threat

**DOI:** 10.1038/s41598-025-94580-3

**Published:** 2025-03-20

**Authors:** Stefan G. Hofmann, Christoph Vogelbacher, Verena Schuster, Marlon Westhoff

**Affiliations:** Department of Psychology, Philipps-University Marburg, Schulstraße 12, 35032 Marburg, Germany

**Keywords:** Network analysis, GIMME, DCM, Thought suppression, Brain network connectivity, Neural correlates, Psychiatric disorders, Neurological disorders, Brain imaging

## Abstract

Suppressing upsetting thoughts can cause psychological distress but might also enhance mental health when used flexibly to suppress the imagination of future threat during challenging times. To investigate the neural correlates of suppressing and imagining future threat, a cohort of 65 participants underwent a previously examined "Imagine/No-Imagine" paradigm while examining brain activation using magnetic resonance imaging. We observed activity of the inferior frontal gyrus, middle frontal gyrus (MFG), superior parietal lobule, and superior occipital sulcus during thought suppression, whereas imagining future threat elicited activation in the bilateral posterior cingulate cortex (PCC) and ventromedial prefrontal cortex (vmPFC). Subjective levels of anxiety, stress, and depression as covariates did not alter these results. To further examine the group and individual-level network dynamics, we conducted dynamic causal modeling (DCM) and group iterative multiple model estimations (GIMME). The DCM model showed that during suppression, the MFG positively influenced the vmPFC and right PCC. In contrast, the vmPFC and the left and right PCC showed positive connections to the MFG during imagining. This suggests that the neural correlates of self-regulation involve an information flow between the PCC and the PFC. In addition, GIMME identified group-level connections between the right and left PCC and between the left PCC and vmPFC, reflecting the information flow during suppression and imagination of future threat, respectively. Considerable interindividual heterogeneity in the connectivity patterns became apparent, pointing to the existence of different biotypes.

## Introduction

Thinking about future uncertainty is known to evoke distress^1^, and excessive future speculation may even be fundamental to psychological conditions, such as anxiety disorders^2^, and post-traumatic stress disorders^3^. The imagination of future fears also occurs more strongly in anxious individuals^4,5^. There are different coping strategies to deal with the imagination of future fears, including mindfulness approaches, that shift the attentive, non-judgmental focus to the “here and now” experiences or suppressing distressing thoughts to avoid this experience. Both approaches downregulate the affective responses to these thoughts^6^.

Historically, suppressed content persists in the unconscious mind and resurfaces indirectly and has been viewed as maladaptive. Recent research indicates that training to suppress upsetting thoughts may actually foster mental health during challenging circumstances. Addressing potential future threats can thus significantly affect an individual’s mental well-being. Therefore, it is crucial to adapt established strategies, such as the suppression of imagining future threats, to enhance mental health^7^. Additionally, it has been documented that consistent suppression of imagining future threats reduces their intrusive nature^8^ but depends on the stress level of a person^9^.

Thought suppression and memory control have been investigated with the Think/No-Think Task (TNT;^10^). During this task, participants are instructed to either think about the shown target word (Think condition) or to actively suppress thoughts connected with the shown target word (No-Think condition). Several functional magnetic resonance imaging (fMRI) studies have been investigating the underlying neural correlates of suppressing memories in the brain using the TNT paradigm^11^. Suppression is associated with activation in medial prefrontal, striatal, and frontoparietal intrinsic connectivity networks as well as a downregulation of the hippocampus (HC). More specifically, suppression increases activity in the right dorsolateral prefrontal cortex (dlPFC) and decreases activity in the HC^12^. The dlPFC is exerting top-down inhibition on crucial retrieval processes in the HC, which impede the reactivation of unwanted memories, given the HC’s role in encoding these. Additionally, the parahippocampal cortex (PhC), associated with the retrieval of detailed and vivid memories, is often downregulated^11^.

To investigate the neural correlates of suppressing and imagining future threats, Benoit et al.^13^ developed and tested the Imagine/No-Imagine Task (INI). During this task, participants were instructed to either imagine an unpleasant scenario (Imagine condition) or to actively suppress thoughts about the scenario (No-Imagine condition). Suppression engaged the right dlPFC, whereas the HC and the ventromedial prefrontal cortex (vmPFC) exhibited reduced activation compared with Imagine trials. Furthermore, the results suggested that suppression was less effective in anxious individuals.

The aim of this study was to investigate the brain regions involved in the suppression and imagination of future threats by adapting a specific task (INI-task) for fMRI utilized by Benoit et al.^13^. This adaptation was designed to meet current neuroscience data standards using various BIDS-apps^14^ and open-source software. We hypothesized that suppression would activate the right dorsolateral prefrontal cortex (dlPFC), hippocampus (HC), and ventromedial prefrontal cortex (vmPFC), as previously suggested by Benoit et al.^13^. Additionally, we aimed to provide support for the stability of this paradigm. To achieve this, we employed a data-driven approach to assess the paradigm’s robustness without relying on any a priori definitions of the regions. Our expectation was that this approach would support the stability of the paradigm, demonstrating that the same regions would consistently be activated. In addition, we examined the network dynamics to analyze connectivity between brain regions, aiming to examine connectivity patterns based on their time series^15^. Such network analytic approaches can uncover topological properties that are still not found in the structure and function networks of human brains and identify the information flow^16^. Accordingly, we employed Dynamic Causal modeling (DCM;^17^) and Group Iterative Multiple Model Estimation (GIMME;^18^) to examine the connectivity patterns among brain regions involved in suppression and imagination mechanisms.

## Method

### Participants

A total of 65 participants were recruited (42 female and 23 male) with a mean age of 24.23 years (*SD* = 2.41; range = 21–31). Participants were not color-blind and reported no history of psychiatric disorder. All participants had no contraindication for MRI. All participants gave written informed consent as approved by the local Ethics Committee (Number 2022-22v-Amendment). For the analysis, 6 participants had to be excluded, due to excessive motion, defined as movement exceeding a 3 mm threshold either during or between MRI runs. Five participants did not show individual peaks (a cluster of 4 voxels) within the group mask for any of the four extracted regions. Consequently, they were excluded from the analysis, resulting in a final sample of 54 participants (33 females/21 males, mean age: 24.19 years (*SD* = 2.29, range 21–31).

### MRI settings

The MRI assessments were conducted utilizing a 3 T MRI scanner (Tim Trio, Siemens, Erlangen, Germany), equipped with a 32-channel head matrix receive coil. First a magnetization-prepared rapid gradient-echo (MPRAGE) structural image (time of repetition (TR) = 1900 ms; time of echo (TE) = 2.52 ms; flip angle = 9°; field-of-view (FOV) = 256 mm × 256 mm × 176 mm; 1 mm^3^ isotropic voxels) was acquired. For the task, the acquisition of T2*-weighted echo planar images (TR = 1 s; TE = 30 ms; flip angle = 70°; FOV = 192 mm × 192 mm; 3 × 3 × 3 mm^3^ voxels; interslice gap = 25%; 32 slices obtained in descending order; 278 volumes for each run, including five dummy volumes) were implemented. MRI data quality was checked by using a stringent protocol for ensuring the temporal stability of the MRI scanner, involving weekly phantom measurements by the MRI center.

### Data acquisition procedure

The experimental protocol was conducted over two consecutive days. On day one, participants generated and rated 18 personal fear-provoking scenarios. Participants were asked to vividly imagine a fear-provoking scenario and provided a short description of the event to verify compliance with the rules. For each episode, they also provided a reminder word as an obvious cue for the specific fear, and a code word representing a typical aspect of their imagination that would remind them of the respective episode, which could not be part of its description. They then rated those scenarios on a five-point scale with regard to various criteria: vividness of typical imagination, emotional intensity, the likelihood of occurrence, temporal distance in the future, and frequency of thought. Following Benoit et al.^13^, these scenarios were randomized into three conditions (imagine, suppress, baseline), each containing six words, based on these ratings. The randomization included the requirement that the conditions could not differ in terms of the rated criteria for each individual, ensuring that the conditions were comparable. This was done using a conservative homogeneity criterion, with non-significant differences between conditions indicated by a *p*-value above 0.1 (using an ANOVA; see supplementary materials). Differences between the conditions below *p* = 0.1 would therefore be significant and would not be allowed by the randomization process. The words were subsequently divided into two sets. The first set, referred to as the training set, contained one word from each category. The second set, designated as the MRI set, comprised the remaining words from all conditions. Notably, the baseline stimuli were only utilized further during the recall phase following the MRI measurement. To measure subjective levels of anxiety and related constructs, subjects also completed the Depression, Anxiety and Stress Scale (DASS-21;^19^). On day two, participants underwent the Imagine/No-Imagine phase (see fMRI task). Data collection ended with a recall phase, in which participants were asked to identify the previously generated scenarios. Participants were shown the hint words of the baseline stimuli on a computer for a period of 4 s, accompanied by a 400 ms inter-stimulus interval. The participants’ task was to verbally identify the corresponding code word for each presented hint word within this 4-s timeframe. This recall phase was pivotal in assessing the participants’ memory and association abilities related to the scenarios generated during the fMRI task. Differing from the original design^13^, the free simulation phase and the apprehensiveness assessment phase were excluded, as the focus of this study was primarily on the general process of suppression and imagination rather than on the exact content of the thoughts (see^20,21^). Free simulation and apprehensiveness were the final phases of the original paradigm. To maintain comparability with Benoit et al.^13^, we deliberately kept the process identical up to this point.

The study was approved by the Ethics Committee of the Department of Psychology of the Philipps-University of Marburg (Reference: 2022-22v). All methods were performed in accordance with the relevant guidelines and regulations. All procedures are in accordance with the 1964 Helsinki declaration and the Guideline for Good Clinical Practice.

### fMRI task

On the second day of the study, MRI measurement was conducted. Preceding the MRI scanning, participants engaged in a preparatory training session outside the scanner. This session involved task performance utilizing a training set, designed to acclimate participants to the task requirements and address any unresolved queries. During the training, participants were exposed exclusively to words from the “imagine” and “suppress” conditions. Upon completion of the training, participants proceeded to undergo MRI scanning.

In the MRI scanner, the initial step involved acquiring a localizer and an AAH scout scan, followed by a T1-weighted (T1w) structural imaging measurement. During this structural imaging, the monitor remained inactive to prevent any visual distractions for the participants. After the structural imaging, participants underwent a second training session within the MRI scanner. For this intra-scanner training, the same set of training words used in the first external session was utilized. Notably, during this phase, the MRI scanner was not operational, focusing solely on acclimating the participants to the task within the scanning environment.

**Fig. 1:**
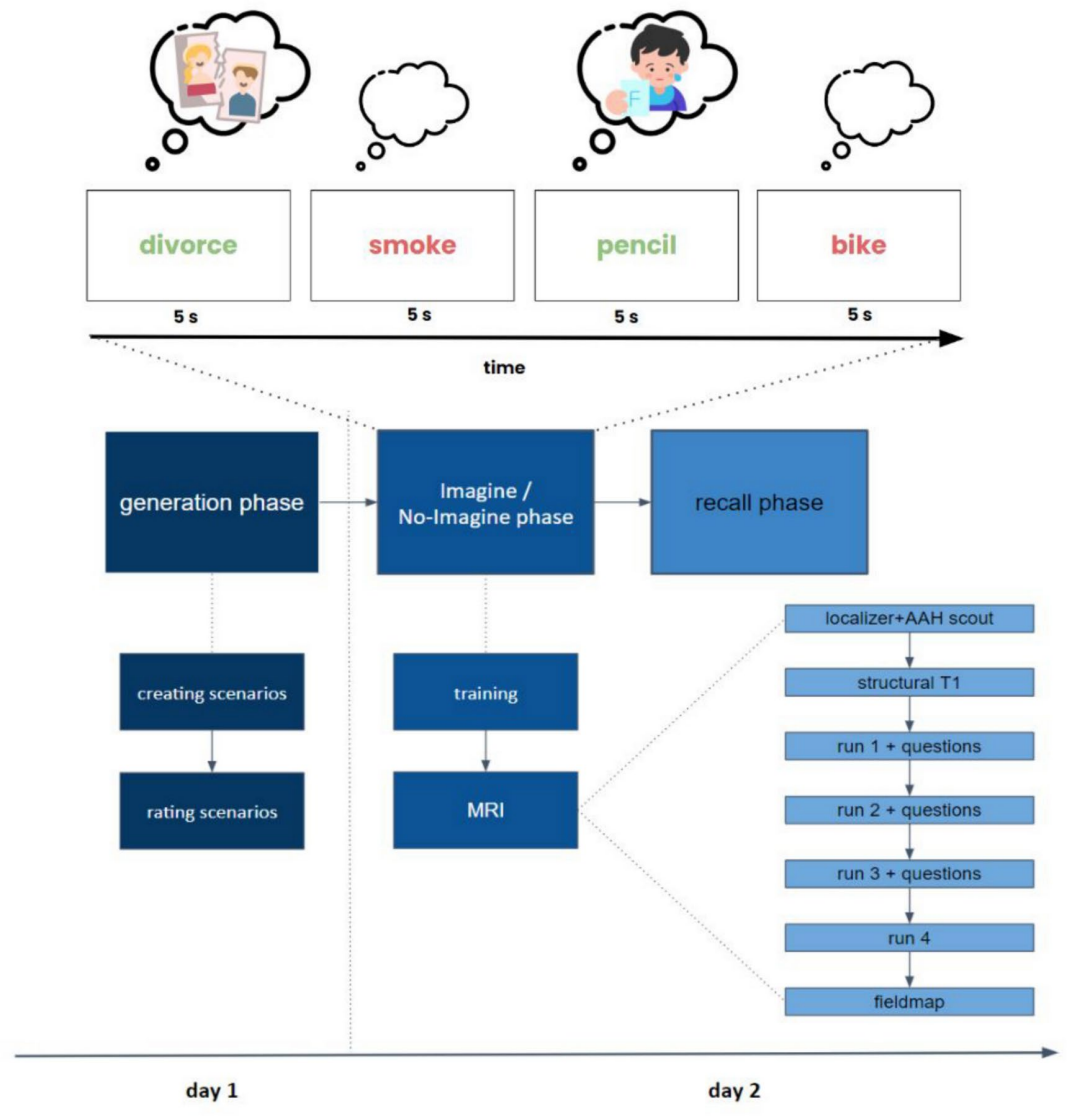
The imagine/no-imagine task (INI) paradigm. The experimental protocol spanned two consecutive days. On the first day, participants generated and rated 18 personal fear-provoking scenarios. For example, the participant might describe sitting at the kitchen table in the evening when their partner announces they want a divorce. They could elaborate on how emotions like anger and sadness emerge, and how tears start to well up in their eyes. For each scenario, they provided a reminder word, i.e., ‘divorce’ as an obvious cue and a code word representing a typical aspect of their imagination, i.e., ‘glass’. They rated these scenarios on a five -point scale. On the second day, MRI measurements were conducted. Before scanning, participants underwent a preparatory training session outside the scanner to familiarize themselves with the task. Participants repeatedly imagined events associated with green reminder words while trying to suppress any thoughts related to episodes with red reminder words. During the MRI scan, the protocol began with a localizer and AAH scout scan, followed by T1-weighted (T1w) structural imaging. The fMRI task included four blocks of 30 words each, displayed for 5 s with a variable inter-stimulus interval (1.5 to 7.5 s, averaging 2.5 s). Diagnostic questions were asked between runs to ensure task adherence. After the task runs, a field map was acquired, and data collection concluded with a recall phase where participants identified the previously generated scenarios.

The fMRI task utilized an event-related design and consisted of four blocks, each containing 30 words. Words were displayed in either red for suppressing or green for imagining for a duration of 5 s in the middle of the screen, interspersed with a variable inter-stimulus interval (jitter) ranging from 1.5 to 7.5 s, averaging 2.5 s with a standard deviation of 1.2 s. The conditions were randomized such that no more than three consecutive words could belong to the same condition. Between the runs, diagnostic questions were asked to ascertain the participants’ adherence to the task instructions. After completion of the task runs, a field map was acquired. See Fig. 1 of an example of the fMRI paradigm.

### MRI data analyses

MRI data was transformed from the Digital Imaging and Communications in Medicine (DICOM) format into the Brain Imaging Data Structure (BIDS) format using heudiconv (version: 0.12.0;^20^). For checking the quality of the data, the tool MRIQC (version 20.0.6;^21^) was used to identify signal inhomogeneity, movement, and artifacts. The preprocessing of the fMRI data was performed by using the standard pipeline of FMRIprep (Version 20.2.7;^22^).

For the fMRI data analysis, a GLM approach was used. Therefore, a BIDS model was created to run the first-level analysis by using fitlins (version 0.10.1;^23^) on the preprocessed data. The movement parameters from preprocessing (including the translation and rotation parameters, each with three degrees of freedom, and framewise displacement) were included as regressors, along with the first six additional noise components calculated using anatomical CompCor. Also, a high pass filter of 0.008 Hz and smoothing of 8 mm (full-width half maximum, FWHM) were applied. For each participant, the contrasts suppress > imagine and imagine > suppress were calculated. Contrasts were created to identify brain regions specifically associated with each condition. This approach reveals differences between the two conditions by highlighting regions that play a comparatively prominent role during suppression and imagination, rather than merely identifying areas that are active during these processes. Thus, contrasts (e.g., suppress > imagine) of the brain regions reveal only relative differences between conditions, as both can exhibit positive or negative activation. The second level analysis was performed using the tool nilearn (version: 0.10.2;^24^).

First, we used the z-standardized single subject contrasts suppress > imagine and imagine > suppress, including sex and age as covariates, to perform a one-sample *t*-test including an FWE < 0.0.05 in line with Benoit et al.^13^ but did not detect activation within the hypothesized regions. Next, we applied a threshold of FPR < 0.001 and a cluster threshold of 10 voxels to mitigate the Type II error rate. Cluster sizes were validated using AFNI’s 3dClustSim (see Supplementary Materials)^27^. Given that we applied different thresholds, which increased the risk of false positives, we then conducted a control analysis to mitigate this risk. We corrected our results for multiple comparisons by adjusting the alpha threshold, dividing it by 2, which is the number of conducted analyses. Then, a region extraction for the contrasts of suppress > imagine and imagine > suppress was performed to find the cluster of at least 19 voxels. The resulting regions were used as a mask to extract the time series.

A two-step procedure was employed for the selection of regions of interest (ROI), following the analytic strategy of Benoit et al.^13^, as well as approaches used in previous studies (e.g.,^28–30^). Initially, a 12-mm spherical region was centered around the peak activation of every ROI identified from group-level analysis to identify the most representative point of activation for each individual. This approach ensured that the main point of activation was included and is based on a broader dataset, thus minimizing individual bias. Following this, a 6 mm spherical region was centered on the newly defined peak to refine the localization of activation and identify more precise, functionally relevant points for the final ROI. This specific method is crucial for the accuracy of DCM analysis, because precise localization of activation points is essential for accurately modeling neural dynamics. Similar approaches have been employed in previous studies, including larger initial ROIs followed by refinement, helping to account for variability and ensure robust peak identification^28,30,31^. This sphere was used for the extraction and also the z-standardization of time series data from the preprocessed fMRI data acquired using the fmriprep pipeline.

For a supplementary investigation into whether anxiety and related constructs contribute to these results, we incorporated the three subscales of the DASS-21, namely anxiety, depression, and stress. We utilized linear regression analysis to examine the association between the activated brain regions in terms of the z-standardized contrasts of the condition suppress > imagine and the three subscales of the DASS-21.

### Network analyses

We adapted the MR parameters for the INI task of Benoit et al.^13^ with modifications such as a shorter TR (1 s instead of 2 s), enhancing temporal resolution for better monitoring of brain activity. The reduced TR likely improved effect detection by providing a greater number of data points for either suppressing or imagining future threats and has also been an improvement for the network analyses.

Investigating the underlying neural correlates during the suppression and imagination of future threats, we used two network approaches (DCM and GIMME). Based on the results of the regional activation, time series data were extracted for the ROI, including the left and right posterior cingulate cortex (PCC), the ventromedial prefrontal cortex (vmPFC), and the middle frontal gyrus (MFG), as Benoit et al.^9^ (the IFG, SPL, and SOS were excluded due to their association with language attention). Time series data were used for the network analyses, incorporating both conditions (i.e., suppression and imagine) as the contrast values (i.e., relative differences between the conditions) reflect the activation/deactivation of both. The modulation focus, following Benoit et al.^13^, was on the suppression condition. Thus, for both the DCM and GIMME analyses, input modulation was applied to the suppression task. In the case of GIMME, input modulation to the suppression task means that the binary task vector of the exogenous variable (i.e., condition) is coded as 1 for suppression and 0 for imagine. For both network analyses, the same time series of the ROIs were used, instead of using the integrated DCM time extraction routine, to make both methods more comparable.

To estimate the connectivity, DCM, as implemented in SPM12 (version v7771, www.fil.ion.ucl.ac.uk/spm/), was used in combination with MATLAB (version R2024a, mathworks.com). For model fitting, we used the extracted time series, adjusting the parameters to enhance the free-energy estimate for the model evidence. More specifically, we developed 63 different models based on the four ROIs, each a variation of a core model that included the MFG, left and right PCC, and the vmPFC as nodes, featured inhibitory auto connections within each region, and allowed bidirectional intrinsic connections among all regions. The variations across these models included changes in (i) the direction of connectivity modulation during the task (bottom-up, top-down, or bidirectional), (ii) the region hypothesized to undergo modulation (left and right PCC, vmPFC, MFG, or a combination of all), and (iii) the origin of the driving input (either left and right PCC and vmPFC or MFG, or a combination of all). These models were then categorized into different families (top-down, bottom-up, and bidirectional), and Bayesian Model Selection (BMS) was applied using a random-effects framework. BMS assesses the exceedance probability, which measures the likelihood of a particular model family being more suitable than others in explaining the data from a random participant, taking into account the complexity of the model. Following this, Bayesian Model Averaging (BMA) was used to evaluate the modulatory effects and effective connectivity (the aggregate of intrinsic and modulatory connectivity) in the most plausible models. BMA calculates the weighted averages for each model parameter, with the weights based on the posterior probability of each model.

GIMME^18^ was applied as a second connectivity method, unveiling general connections among ROIs while taking into account individual differences. It employs a unified structural equation model (uSEM;^32^) to identify both generalizable and non-generalizable relationships among ROIs, acknowledging the heterogeneity within a given population. Through an iterative approach, it selects contemporaneous and lagged paths for individuals, if they improve their model fit. Subsequently, these paths are estimated for the group model if a sufficient number of individuals have the path^33^. GIMME therefore indicates common connections between ROIs in the group (i.e., group-level connections), as well as connections between ROIs that only individuals have (i.e., individual differences). By employing these integrated pathways, GIMME identifies typical connections among ROIs’ components within a population to distinguish signal from noise^33^. Unlike conventional approaches that treat individual differences as estimation errors in group averages, GIMME assumes shared connectivity patterns among individuals, enhancing the accuracy and effectiveness of its inference procedure. By considering within-person variability, GIMME derives individual models and extends these findings nomothetically. By integrating causal structures at both individual and group levels, GIMME enables more precise generalizations about connectivity patterns within the studied population using idiographic data^33^. By taking individual differences into account, individual networks could be modeled. However, the focus of the paper was on the bottom-up derivation of generalized connections that take individual-specific connections into account.

The extension HRF-GIMME enables the assessment of task-related effects on functional connectivity networks in fMRI data by modeling the direct and modulatory task effects through the estimation of the hemodynamic response function (HRF;^34^). Direct effects of a task can be understood as the extent to which a task influences the variability of the blood oxygenation level-dependent activity (BOLD) of an ROI. Modulating effects include the influence of task stimuli on the strength or direction of the relationships between two ROIs. The HRF, which describes the expected time course of changes in deoxyhemoglobin that occur after neuronal activity, is different for individuals and thus requires deriving person-specific HRF parameters^17,35^. Therefore, HRF-GIMME uses a smoothed finite impulse response function (sFIR;^36^) to estimate the HRF for each individual. For this analysis, the exogenous stressor was set on the time points when participants had to suppress. The probability of detecting an effect for the group was set at 75%.

## Results

### Recall

The literature suggests that future threats are less present after performing a suppression task compared to imagining those threats. A recall of these threats is performed after the INI task to investigate the success of suppression. Our results indicate no significant difference in recall rates between suppressing (*M* = 84.75%; *SD* = 17.86) or imagining (*M* = 81.36%; *SD* = 20.31) future threats or baseline (*M* = 81.92%; *SD* = 20.13). An ANOVA comparing the three conditions yielded non-significant differences (*F*(2, 174) = 0.42, *p* = 0.66).

### Brain activation

**Fig. 2:**
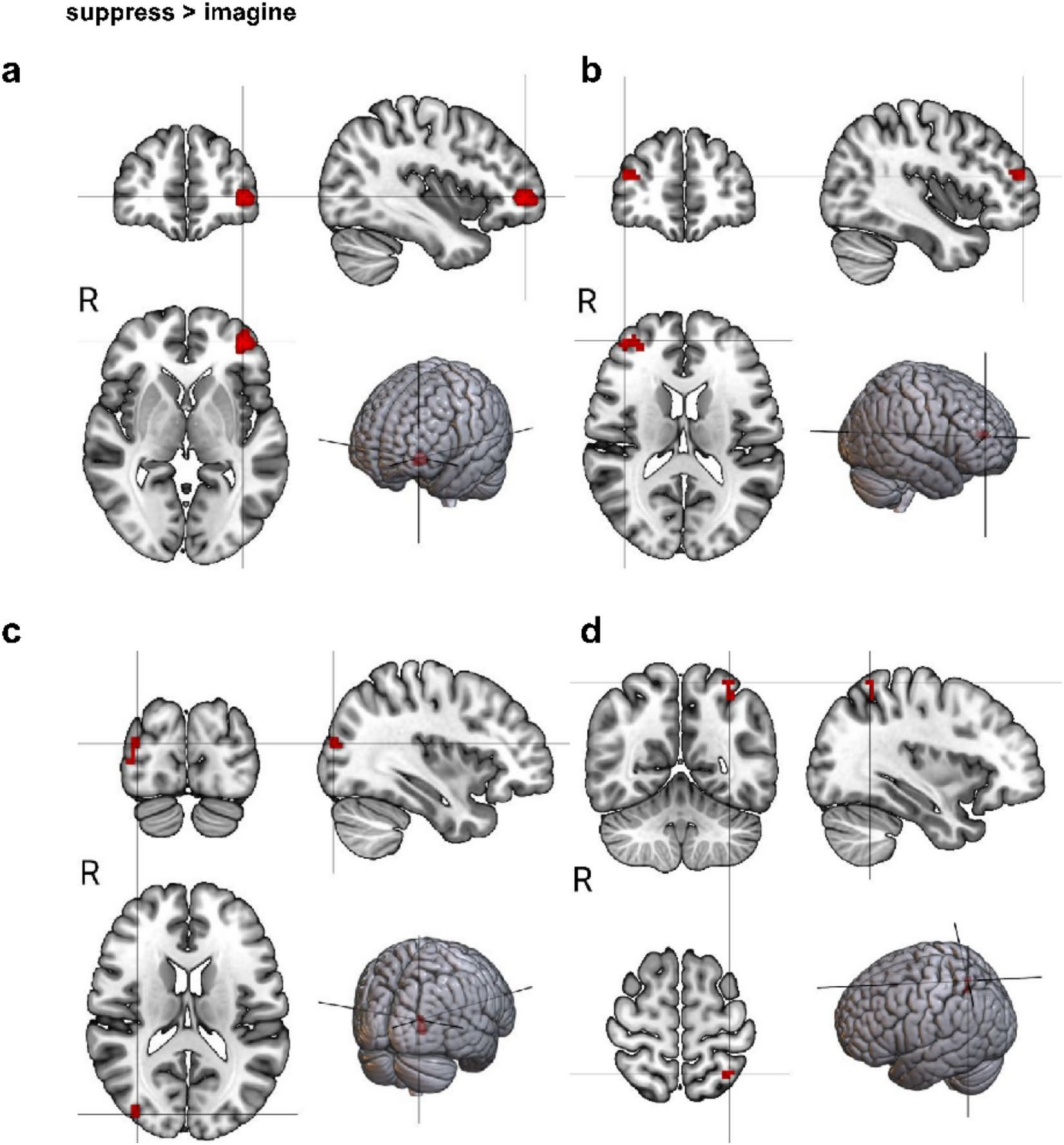
Each region that survived the FPR threshold for the condition suppress > imagine; (**a**) IFG = inferior frontal gyrus, (**b**) MFG = middle frontal gyrus, (**c**) SOS = superior occipital sulcus, (**d**) SPL = superior parietal lobule.

**Fig. 3:**
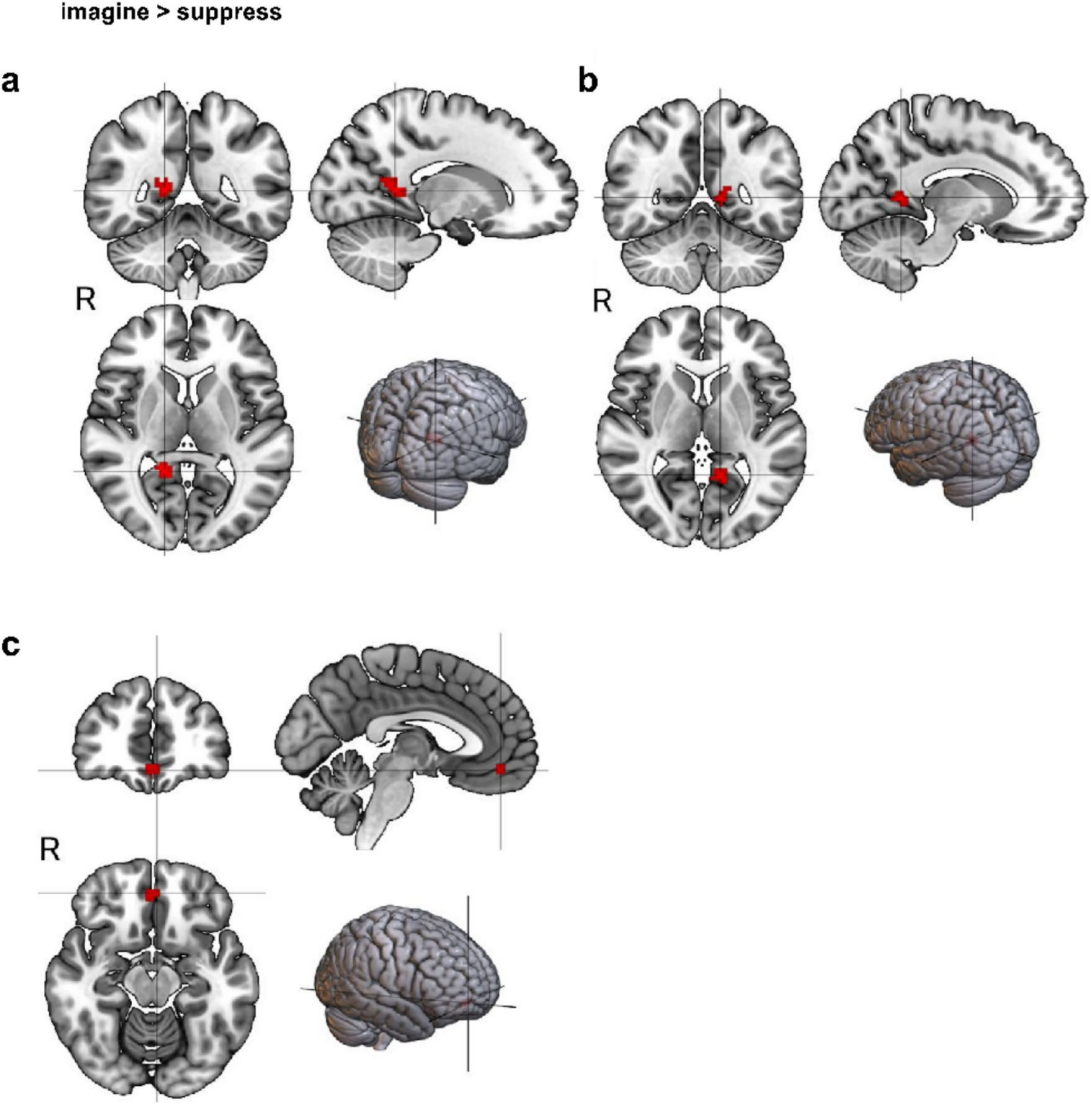
Each region that survived the FPR threshold for the condition imagine > suppress; (**a**) right PCC, (**b**) left PCC, (**c**) vmPFC.

To identify the involved brain regions during suppression and imagining future threats, we used a general linear model (GLM). Applying a false positive rate (FPR) < 0.001 correction and cluster correction with a threshold of 10 voxels (the results for each condition are shown in Table 1). In the suppress > imagine contrast (see Fig. 2), significant clusters emerged in the inferior frontal gyrus (IFG), middle frontal gyrus (MFG), superior parietal lobule (SPL), and superior occipital sulcus (SOS). For imagine > suppress (see Fig. 3), clusters were found in the bilateral posterior cingulate cortex (PCC) and vmPFC. No hippocampus clusters were detected, compared to the findings of Benoit et al.^13^. These findings were consistent with our control analysis (FPR with *p* = 0.0005, cluster threshold of 10 voxels), as presented in the Supplementary Materials.

**Table 1:** Each region that met the FPR threshold for the 'suppress > imagine’ and the ‘imagine > suppress’ condition. It includes information on the hemisphere, cluster size, the average z-value of the region, and its MNI coordinates. IFG = Inferior frontal gyrus, SOS = Superior occipital sulcus, MFG = Middle frontal gyrus, SPL = Superior parietal lobule, PCC = posterior cingulate cortex, vmPFC = ventromedial prefrontal cortex.

Suppress > imagine				MNI coordinates		
Region	Hemisphere	Cluster size	Mean z-value	x	y	z
IFG	Left	61	3.87	− 38	47	0
MFG	Right	38	3.44	38	49	16
SOS	Right	35	3.41	38	− 87	7
SPL	Left	22	3.37	− 34	− 54	62

Imagine > suppress				MNI coordinates		
Region	Hemisphere	Cluster size	Mean z-value	x	y	Z
PCC	Left	41	3.33	− 12	− 53	5
PCC	Right	65	3.53	14	− 47	8
vmPFC	Both	25	3.38	0	45	− 12

Supplementary linear regression analysis between the MFG and the respective subscales of the DASS-21, including anxiety (*ß* = 0.34, *p* = 0.48), depression (*ß* =− 0.24, *p* = 0.74), and stress (*ß* = − 0.53, *p* = 0.52), revealed no significant associations (see supplementary material).

### Dynamic causal modeling (DCM)

**Fig. 4:**
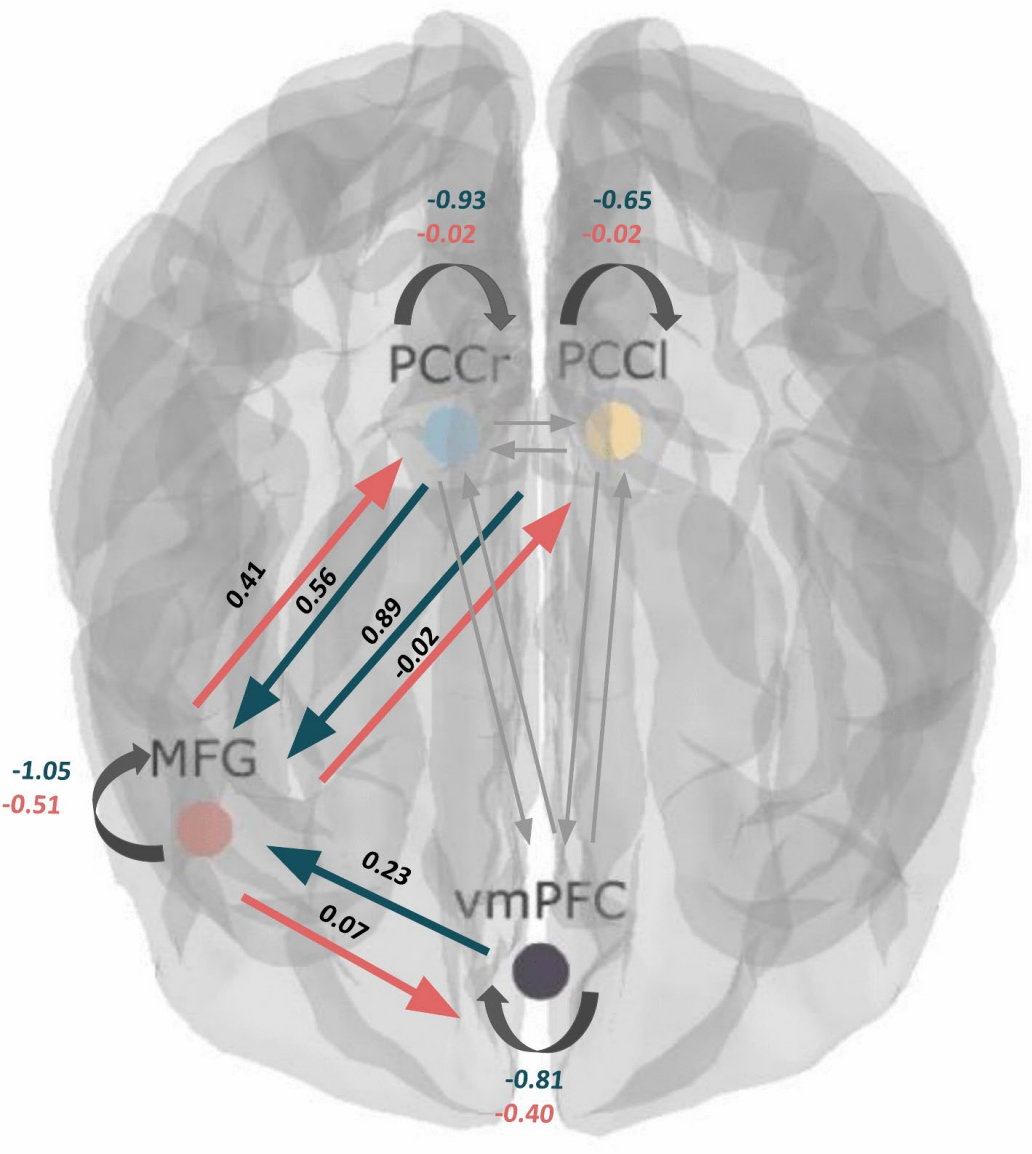
The winning model of the DCM analysis, including the strength of the connection between the regions. For between-region parameters (measured in Hz), positive values indicate excitation, whereas negative values indicate inhibition. Positive values for self-connections represent increased self-inhibition, whereas negative values indicate disinhibition. In the suppress condition (shown in red), the MFG showed positive connections to the PCCr and the vmPFC, with all regions exhibiting disinhibitory self-regulation. In the imagining condition (shown in green), both PCCs and the vmPFC showed positive connections to the MFG with all regions exhibiting negative self-regulation.

For the analysis, 63 models (categorized into top-down, bottom-up, and bidirectional families with variations in the direction of connectivity, modulation, and driving input) were used to calculate the connectivity. The analysis revealed that the bidirectional family was the most successful (expected probability: 89.69%; exceedance probability: 100%). All models were then compared to find the winning model. The winning model (see Fig. 4, expected probability: 13.39%; exceedance probability: 42.54%) showed positive MFG connections to other regions during suppression, with negative autoregulation. During imagining PCCs and the vmPFC maintained positive connections to the MFG with negative self-regulation.

### Group iterative multiple model estimation (GIMME)

Based on the suppression > imagine contrasts, GIMME identified group-level contemporaneous connections between the PCCr and the PCCl, the PCCr, and the vmPFC, as well as a lagged connection between the PCCl and the PCCr at a threshold of 0.75. Each region exhibited a lagged connection to itself, indicating self-regulation.

**Fig. 5:**
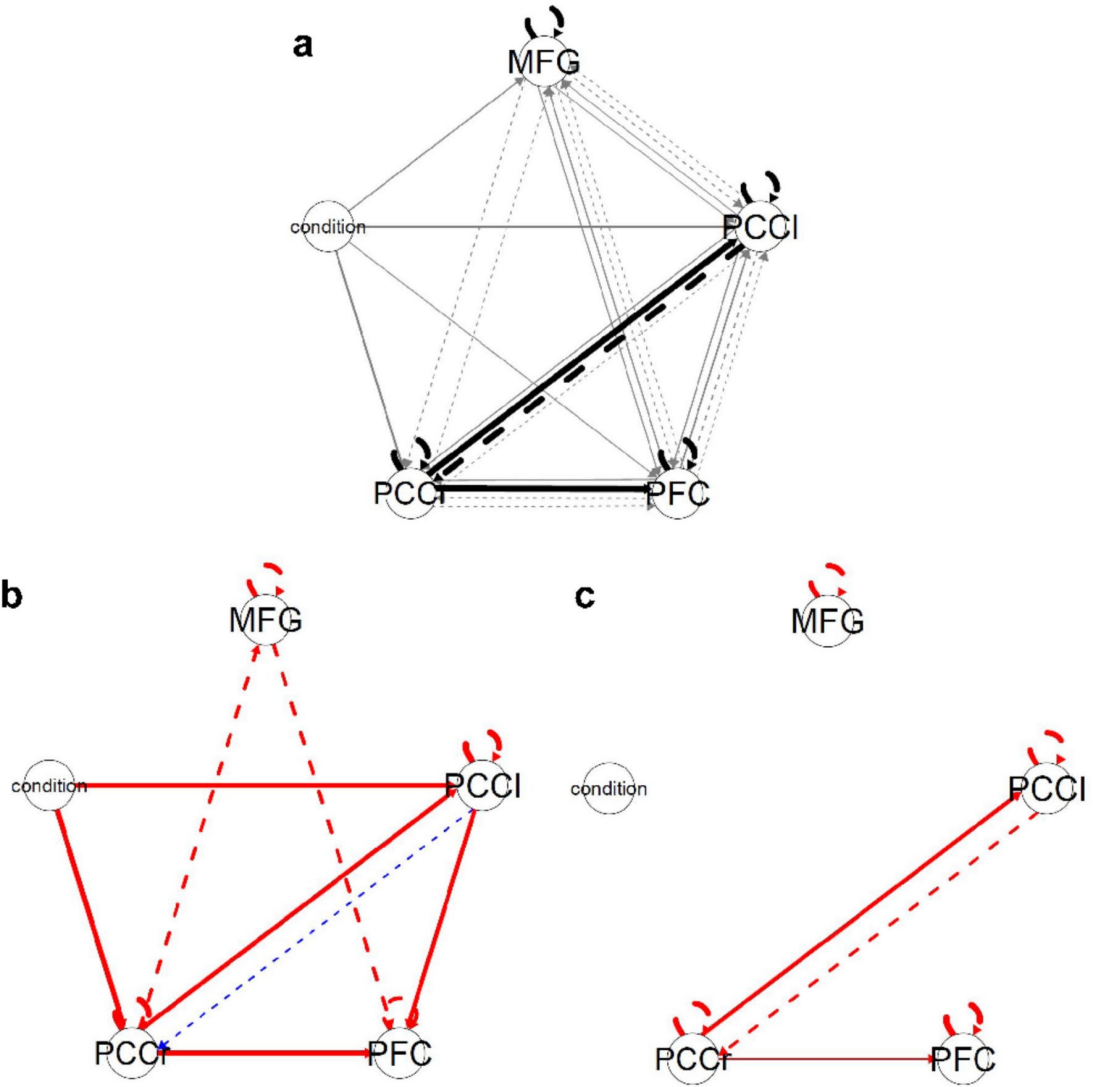
The GIMME network of the included ROIs. In this graph, solid lines represent contemporaneous relations (lag 0), and dashed lines reflect lagged relations (lag 1). The width of paths corresponds to the estimated path weight (corresponds to the count of connections). Black represents group-level paths (for all participants), and gray represents individual-level paths. Condition (Suppress vs. Imagine) is the exogenous variable and is set to the time points where the participants were asked to suppress. Connections indicate an influence of the task condition on the respective ROI. A: shows the results for the group. B and C: Individual results for two exemplary participants (red paths represent positive weights and blue paths represent negative weights).

At the individual level, direct task effects on all ROIs were detected, along with contemporaneous and lagged effects between ROIs, suggesting individual differences in connectivity patterns (see Fig. 5a). Individual networks for two exemplary subjects show positive and negative weighted paths (see Fig. 5b,c).

## Discussion

To investigate the neural correlates of suppressing and imagining future threats, we adapted the INI task by Benoit et al.^13^, used two different network approaches, and an increased sample size to improve statistical power^37,38^. Participants identified feared events with single-word representations.

By applying an FPR correction, we found activation in the bilateral PCC and vmPFC during imagining. The PCC is involved in introspection, memory processes, emotion regulation, and self-awareness^39,40^. The vmPFC plays a pivotal role in regulating emotions^41,42^ and influences fear perception, emotional responses^43,44^, and stress-induced emotion regulation^41^. These regions and their functions are consistent with the imagination of future fears.

We further observed activation in the right MFG, right IFG, SPL, and SOS during suppression. The right MFG serves as a critical hub for integrating dorsal and ventral attention networks, enabling it to interrupt ongoing internal attentional processes and redirect focus toward external stimuli^45^. Meta-analyses focusing on cognitive reappraisal of emotions have consistently identified the right MFG as a central component involved in emotion regulation during cognitive tasks^43,46^. These findings suggest that the right MFG plays a crucial role in shifting attention from external to internal control and in regulating emotions. The right IFG plays a role in various cognitive functions, including attention, motor inhibition, imagery, social cognition, and speech processes^47^. It responds to increased control demands during word comprehension^48^ and tasks requiring inhibition and restructuring of information for efficient sentence comprehension^49^. It is also involved in the implementation of reappraisal strategies^50^. The SPL plays a crucial role in attention and executive control networks, coordinating attention in situations with competing stimuli and facilitating voluntary attentional orienting^51^. It is also associated when subjects have to disengage their attention from fixation and move it to a cued location^52–55^. The SOS is associated with the processing of visual information and is capable of analyzing and synthesizing visual stimuli. The occipital lobe, a visual processing center, is primarily involved in complex visual perception processes^56^. Recent studies have shown that the occipital cortex is subordinated to the attention network, which prioritizes the content of received information and processes images selectively^57^. This suggests that visual information processing in the occipital cortex plays a key functional role in selective attention. Furthermore, the activation observed in the IFG, SPL, and SOS aligns with participants focusing on displayed words, particularly during suppression.

The general pattern of results is consistent with the ones reported by Benoit et al.^9^. However, in contrast to Benoit et al.^13^, who found poorer recall of suppressed fears compared to baseline, our study did not reveal a significant difference. Additionally, unlike Benoit et al.^13^, we did not obtain significant results in the hypothesized brain regions with p < 0.05, whole-brain FWE-corrected. We then applied a more liberal threshold of p < 0.001 using FPR to identify patterns that might have been missed under the strict FWE correction. The decision to use an FPR < 0.001 was made to balance the risk of Type I and Type II errors. While FWE is highly stringent and minimizes Type I errors, it can be overly conservative, leading to an increased risk of Type II errors and potentially missing meaningful activations. We applied cluster correction with a cluster threshold of 10 voxels, which is an additional correction method in fMRI analysis (for a brief discussion, see^58^). Additionally, we performed the recommended analysis and divided the *p*-value of the second analysis by 2 (i.e., Bonferroni corrected). We applied a *p*-value of 0.001/2, i.e., p = 0.0005 using FPR and a cluster threshold of 10 voxels.

The absence of the free simulation and apprehensiveness assessment phase to verify the accuracy of the scenarios might have contributed to the disparity in results. This issue could be resolved in future studies by performing the recall directly before and after performing the task^11^.

We also found no significant association between subjective anxiety or related constructs and the MFG activated during suppression. It should be noted, however, that our methodology differed from Benoit et al.^13^. Instead of the State-Trait Anxiety Inventory Form Y (STAI-Y), we used the DASS-21, to examine whether anxiety uniquely contributes to the results. Moreover, Benoit et al.^13^ linked anxiety with future apprehensiveness through self-reports, whereas we examined the influence of anxiety on the associated activation of brain regions during suppression with anxiety. Therefore, the results regarding subjective anxiety cannot be directly compared between Benoit et al.^13^ and our study. Future studies should examine whether this pattern of results holds true in individuals with clinical levels of anxiety because it is quite possible that the impact of anxiety and related constructs on the neural correlates of threat processing needs to succeed a critical level to show an effect.

One could argue that our exclusion criteria artificially inflated false positive rates (see^59,60^) as we removed participants who did not exhibit individual peaks (a cluster of 4 voxels) within the group mask for any of the four extracted regions. However, this exclusion was based on predefined data quality criteria and aligns with previous DCM analyses. Studies by Frässle et al.^31^, Stephan et al.^61^ and Seghier et al.^28^ similarly excluded participants who lacked activation in one or more predefined ROIs, as DCM requires consistent region definitions across subjects. Importantly, the absence of a BOLD response does not necessarily indicate a lack of task engagement or poor data quality. Individual differences in neural responses may lead to non-significant effects despite active participation^62^. To enhance assessments of task engagement, future studies could integrate additional behavioral measures, such as eye-tracking or button presses.

For the DCM analysis, we constructed various model families (bottom-up, top-down, and bidirectional) using the identified ROIs. Results revealed the bidirectional family as the winning model with an exceedance probability of 100%. Bidirectional family models distinguish themselves through their modulated connections between regions and input regions, with stronger modulated connections indicating higher probabilities. Notably, the model featuring solely the modulated input on the MFG resulted in the highest probability among all models. Our model indicates that inputting suppression on the MFG positively influences the function of the vmPFC and the PCC, which are primarily responsible for imagining future threats. Thus, suppression primarily activates the MFG, which in turn influences the vmPFC and the PCC, creating a network that supports the active suppression of stressful thoughts. In contrast, imagination, as reflected in the ROI activations, involves these regions in a different capacity, facilitating the simulation of future threats. The connections identified in our DCM analysis suggest that while suppression modulates specific neural circuits to reduce thinking about future threats, imagination engages these circuits in generating detailed future scenarios. This finding aligns with the results of Benoit et al.^13^, who observed that suppressing triggers activation in the right dlPFC that affects the functioning of the HC and the vmPFC. However, in contrast to the findings by Benoit et al.^13^, we did not detect hippocampus clusters. Future studies will need to examine the reason for this inconsistency.

GIMME results revealed connections between the right and left PCC, as well as the right PCCr and the vmPFC. A lagged effect from the PCCl to the PCCr emerged, indicating a regulating effect of the PCCl on PCCr at the next time point. Lagged autoregressive effects of the ROIs indicate self-regulatory activities. Group-level connections between these ROIs at a group cutoff of 75% indicate valid detection by HRF-GIMME pointing to the information flow during imagination and suppression of future fears. These results suggest that the neural correlates of self-regulation involve an information flow between the right PCC, the left PCC, and the PFC.

The results revealed numerous connections between ROIs at the individual level due to the fact that GIMME considers person-specific connections and estimates each connection weight separately for each individual. These findings point to considerable individual differences in the connections between ROIs in addition to group-level connections, indicating that individuals differ in their underlying mechanisms of imagination and suppression of future fears. Therefore, the use of HRF-GIMME made it possible to detect generalizable group-level connections that exist for a large proportion of individuals while also uncovering individual differences in effective connectivity maps. Accounting for individual differences in connectivity patterns contrasts with traditional methods in fMRI analysis, which often aggregate across individuals by averaging. However, aggregating information across all individuals sometimes results in connectivity patterns at the group level that may not apply to any individual^63,64^. These results raise the possibility for the existence of different biotypes based on the individual connectivity pattern^65^. Future research should examine this paradigm in diverse clinical samples.

## Conclusion

This study provides new insights into the neural correlates of emotion regulation strategies by examining the neural network dynamics of suppressing and imagining future threats. Using the INI task, including adaptations to state-of-the-art acquisition and analysis methods, showed similar active brain areas and neural correlates for suppressing memories or future threats. Suppressing future threats activated the regions IFG, SPL, and SOS that align with participants focusing on displayed words, particularly during suppression, whereas the activation in the MFG shifts attention from external to internal control and regulates emotions. Imagine future threats activated the PCC and vmPFC that describe memory processes as well as emotion regulation.

These results are generally consistent with another, independent research group pointing to the critical role of the middle frontal gyrus in suppressing threat and the involvement of the PCC and vmPFC in imagining threats, but we also observed clear individual differences in the individual connectivity patterns, which will require future investigations into the nature of these individual differences. The DCM analyses showed that inputting suppression on the MFG positively influenced the function of the vmPFC and the PCC. GIMME identified contemporaneous and lagged connections between the PCCr and the PCCl, as well as the PCCr and the vmPFC, which highlights an information flow from the PCC to the vmPFC. However, there was considerable heterogeneity between individuals in the connections between these ROIs, suggesting that individuals differ in their underlying mechanisms of imagination and suppression of future fears. This points to the existence of different biotypes based on the individual connectivity pattern. In addition, the results open the possibility for a clinical intervention by enhancing the suppression training through neuromodulation to improve mental well-being either alone or combined with other treatments, such as exposure therapy and cognitive behavioral therapy^7^.

## Data Availability

All data generated or analyzed during this study are included in this published article (and its Supplementary Information files). The code and all data for this project are also available on the Open Science Framework (OSF) on https://osf.io/4q7d2/.
